# Supplementary material for: Beyond IC50—A computational dynamic model of drug resistance in enzyme inhibition treatment
Source: PLoS Comput Biol. 2024 Nov 7;20(11):e1012570. doi: 10.1371/journal.pcbi.1012570 (PMC11575782; doi:10.1371/journal.pcbi.1012570)
Supplement: S1 Text — Table A shows the system with inactive state binding inhibitors (imatinib and ponatinib), and Table B is for system with active state binding inhibitors (dasatinib). (PDF) [file pcbi.1012570.s001.pdf]

S1 Text: Mass balance equations

change		calculation
$\Delta[\mathrm{E_A}]$	$=$	$((k^{\mathrm{S}}_{\mathrm{off}} + k_{\mathrm{cat}})[\mathrm{E_A S}] - k^{\mathrm{S}}_{\mathrm{on}}[\mathrm{S}][\mathrm{E_A}] + k^{\mathrm{I}}_{\mathrm{trans}}[\mathrm{E_I}] - k^{\mathrm{A}}_{\mathrm{trans}}[\mathrm{E_A}])\Delta t$
$\Delta[\mathrm{E_A S}]$	$=$	$(k^{\mathrm{S}}_{\mathrm{on}}[\mathrm{S}][\mathrm{E_A}] - (k^{\mathrm{S}}_{\mathrm{off}} + k_{\mathrm{cat}})[\mathrm{E_A S}])\Delta t$
$\Delta[\mathrm{E_I}]$	$=$	$(k^{\mathrm{A}}_{\mathrm{trans}}[\mathrm{E_A}] - k^{\mathrm{I}}_{\mathrm{trans}}[\mathrm{E_I}] + k^{\mathrm{R}}_{\mathrm{off}}[\mathrm{E_I R}] - k^{\mathrm{R}}_{\mathrm{on}}[\mathrm{R}][\mathrm{E_I}])\Delta t$
$\Delta[\mathrm{E_I R}]$	$=$	$(k^{\mathrm{R}}_{\mathrm{on}}[\mathrm{R}][\mathrm{E_I}] - k^{\mathrm{R}}_{\mathrm{off}}[\mathrm{E_I R}])\Delta t$
$\Delta[\mathrm{E_{tot}}]$	$=$	$\Delta[\mathrm{E_A}] + \Delta[\mathrm{E_A S}] + \Delta[\mathrm{E_A R}] + \Delta[\mathrm{E_I}]$
	$=$	0

Table A: Table outlining the equations for the change in population of each state in the system for an inactive state binding inhibitor using Euler integration of Equation 2 over time step Δt. The mass balance equation for the change in the total concentration, $\Delta[\mathrm{E_{tot}}]$, is given and shown to be zero.

change		calculation
$\Delta[\mathrm{E_A}]$	$=$	$((k^{\mathrm{S}}_{\mathrm{off}} + k_{\mathrm{cat}})[\mathrm{E_A S}] - k^{\mathrm{S}}_{\mathrm{on}}[\mathrm{S}][\mathrm{E_A}] + k^{\mathrm{I}}_{\mathrm{trans}}[\mathrm{E_I}] - k^{\mathrm{A}}_{\mathrm{trans}}[\mathrm{E_A}] + k^{\mathrm{R}}_{\mathrm{off}}[\mathrm{E_A R}] - k^{\mathrm{R}}_{\mathrm{on}}[\mathrm{R}][\mathrm{E_A}])\Delta t$
$\Delta[\mathrm{E_A S}]$	$=$	$(k^{\mathrm{S}}_{\mathrm{on}}[\mathrm{S}][\mathrm{E_A}] - (k^{\mathrm{S}}_{\mathrm{off}} + k_{\mathrm{cat}})[\mathrm{E_A S}])\Delta t$
$\Delta[\mathrm{E_A R}]$	$=$	$(k^{\mathrm{R}}_{\mathrm{on}}[\mathrm{R}][\mathrm{E_A}] - k^{\mathrm{R}}_{\mathrm{off}}[\mathrm{E_A R}])\Delta t$
$\Delta[\mathrm{E_I}]$	$=$	$(k^{\mathrm{A}}_{\mathrm{trans}}[\mathrm{E_A}] - k^{\mathrm{I}}_{\mathrm{trans}}[\mathrm{E_I}])\Delta t$
$\Delta[\mathrm{E_{tot}}]$	$=$	$\Delta[\mathrm{E_A}] + \Delta[\mathrm{E_A S}] + \Delta[\mathrm{E_I}] + \Delta[\mathrm{E_I R}]$
	$=$	0

Table B: Table outlining the equations for the change in population of each state in the system for an active state binding inhibitor using Euler integration of Equation 2 over time step Δt. The mass balance equation for the change in the total concentration, $\Delta[\mathrm{E_{tot}}]$, is given and shown to be zero.
