# Supplementary material for: Beyond IC50—A computational dynamic model of drug resistance in enzyme inhibition treatment
Source: PLoS Comput Biol. 2024 Nov 7;20(11):e1012570. doi: 10.1371/journal.pcbi.1012570 (PMC11575782; doi:10.1371/journal.pcbi.1012570)
Supplement: S2 Text — These are similar to Fig 3 in the main text. (PDF) [file pcbi.1012570.s002.pdf]

S2 Text: Selection of results for systems with ponatinib and dasatinib

These figures are similar to Figure 3 in the main text.

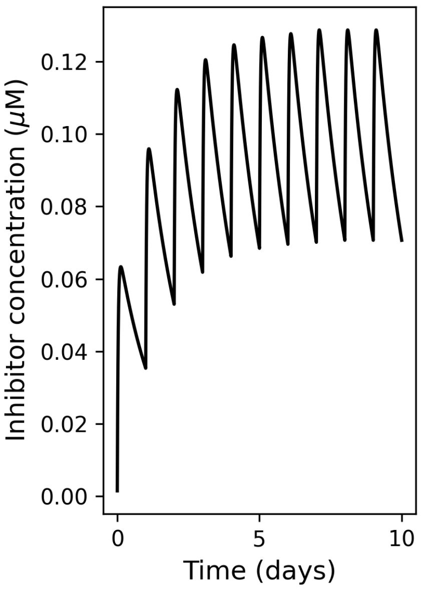

(a) Concentration of ponatinib in the system.

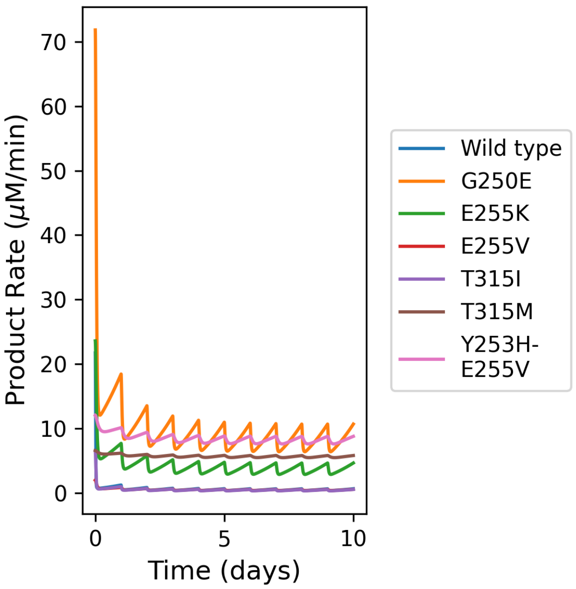

(b) Product formation rates from systems with ponatinib.

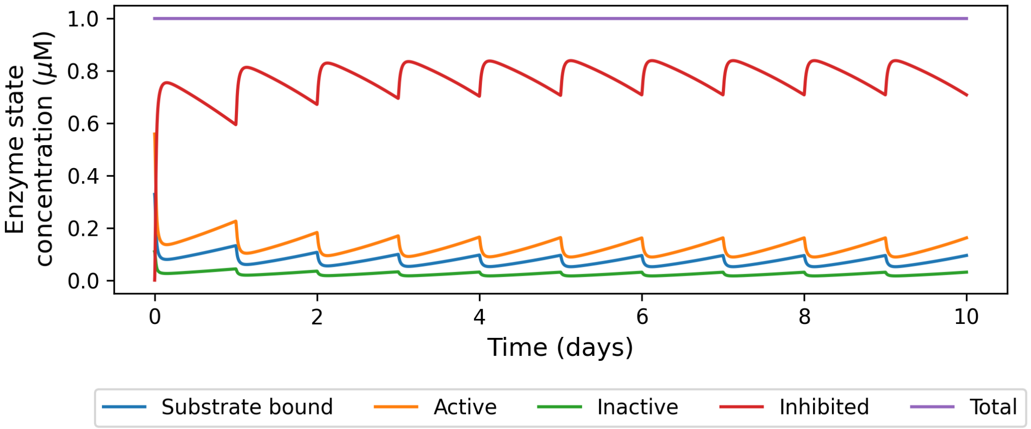

(c) Effect of ponatinib concentration on wild-type states

Figure A: Various outputs from the model with the drug ponatinib. (a) Ponatinib concentrations over the first 10 days of daily treatment doses, calculated as outlined in section 3.1.4. (b) The product formation rates of the wild-type and six mutant Abl1 enzymes over ten days of initial treatment with ponatinib. (c) Effect of Abl1 inhibitor ponatinib on the enzyme states of the wild-type. Here, the growth to steady-state is barely reflected in the enzyme states and the reduction in substrate bound enzymes and increase in inhibitor bound enzymes is almost immediate.

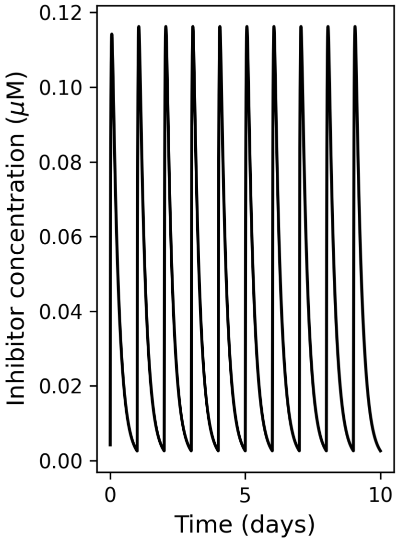

(a) Concentration of dasatinib in the system.

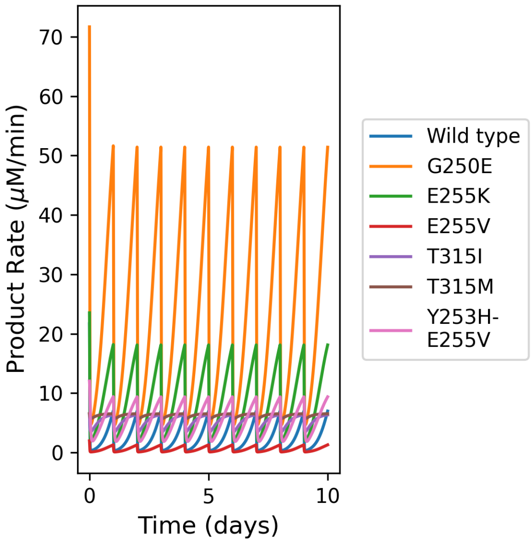

(b) Product formation rates from systems with dasatinib.

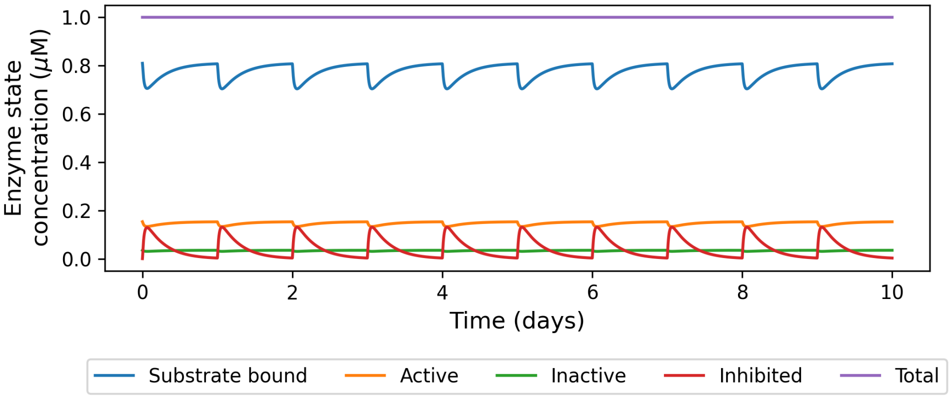

(c) Effect of dasatinib concentration on T315I states

Figure B: Various outputs from the model with the drug dasatinib. (a) Dasatinib concentrations over the first 10 days of daily treatment doses, calculated as outlined in section 3.1.4. (b) The product formation rates of the wild-type and six mutant Abl1 enzymes over ten days of initial treatment with dasatinib. (c) Effect of Abl1 inhibitor dasatinib on the enzyme states of T315I. The change in enzyme states varies, but not by a large amount, indicating that the effectiveness of dasatinib against mutation T315I is low.
