# Supplementary material for: Beyond IC50—A computational dynamic model of drug resistance in enzyme inhibition treatment
Source: PLoS Comput Biol. 2024 Nov 7;20(11):e1012570. doi: 10.1371/journal.pcbi.1012570 (PMC11575782; doi:10.1371/journal.pcbi.1012570)
Supplement: S3 Text — Results as IRP against time for three drug-mutant combinations with random variance in dosing schedule as a test of robustness for the model. (PDF) [file pcbi.1012570.s003.pdf]

S3 Text: A small test of the robustness of the model

To check the robustness of the selected parameters for the model, we simulated a variation in patient dosing. The model is as before, with the exception of the inhibitor concentration calculation. Instead of the exact daily dosing schedule, we implemented a random variation around this time time for each day. The variation was calculated from a Gaussian distributed random number with a standard deviation of 3 hours with the mean value as the start of each 24 hours. Three systems were tested: wild-type Abl1 enzyme with imatinib, G250E mutant with ponatinib, and T315M mutant with dasatinib. The resultant "inhibitory reduction prowess" (IRP) against time for these three combinations with this random variation and without are shown in Figure A. In this figure, the model is shown to be responsive to these variations and does not "overreact" to these changes, the model is therefore robust.

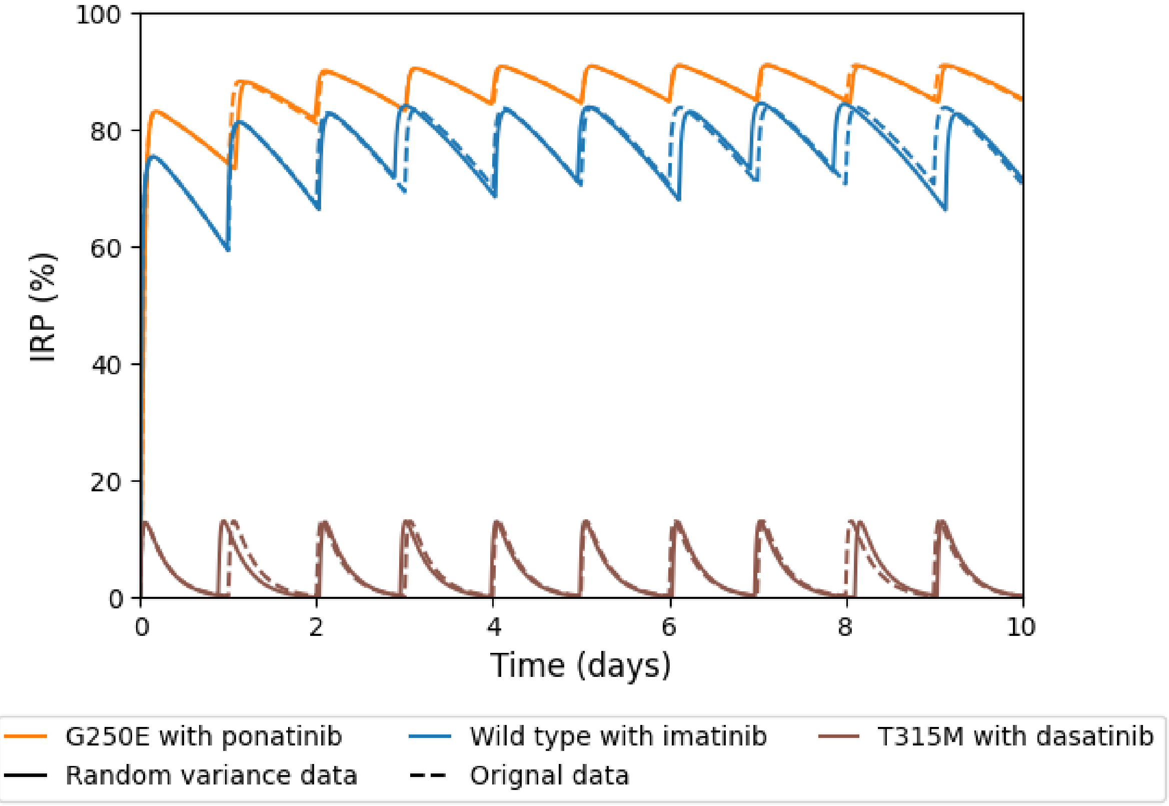

Figure A: IRP against time for the three chosen combinations with and without the random variance in dosing schedule.
