# Supplementary material for: Beyond IC50—A computational dynamic model of drug resistance in enzyme inhibition treatment
Source: PLoS Comput Biol. 2024 Nov 7;20(11):e1012570. doi: 10.1371/journal.pcbi.1012570 (PMC11575782; doi:10.1371/journal.pcbi.1012570)
Supplement: Attachment — Submitted filename: PLOS feedback letter.pdf [file pcbi.1012570.s004.pdf]

Dear editors and reviewers,

Thank you for your consideration and detailed feedback. We have taken your advice in and made the following changes in response to your comments. To give our response clear structure, we have numbered and included the comments we are responding to in italics.

Reviewer 1:

1) *It is not evident that dasatinib cannot bind to the inactive conformation. Does the analysis change if dasatinib is treated as an active and inactive conformation binder?*

We have included references to papers that evidence that dasatinib binds mainly to the active state and changed the wording to reinforce this.

2) *Please clarify why the kon rate is constant for the "active" conformation binders and koff is constant for the "inactive" conformation binders.*

We have reviewed this assumption and rewritten the section titled "Abl1 inhibitor binding rate constants: k^R_off and k^R_on." within section 3.1.3. On reviewing, we closely examined the binding site of the ATP and dasatinib compared with the locations of the mutations, concluding that only T315 mutations will affect the drug binding in the active state, therefore the other mutations will be assigned a similar binding k^R_on for dasatinib as the wild-type. However, the T315 mutations will differ in k^R_on. To find these values we assume a similar change as the substrate binding change from wild-type to these mutations. The general aim of keeping certain rates the same as the wild-type is to keep the simulations tractable, which we now emphasise in the text.

3) *G250E, E255K and E255V may favor (or bias towards) the inactive conformation (and explain why inactive conformation binders are less active) and as such the active state may not be populated as much with these mutations. Should this be taken into consideration in the weighting?*

This is taken into consideration in the weighting with the values of ΔG describing this. We have now emphasised this in the text and in the caption of table 2.

Reviewer 2:

1) *It would be helpful to test the numerical robustness of the system of equations to inaccuracy in the assumed rate constants and any other uncertain parameters. I would suggest applying random variations to individual parameters of a magnitude congruent with the best estimates of their uncertainty and rerunning the differential equation based numerical simulations. From this, one should be able to confirm whether robust or chaotic behaviour is observed. As many repeats as reasonably affordable should be carried out. Averaged results plus uncertainties could then be reported.*

We have added supplementary materials, in which we include a test of robustness of the system by adding a random variance into the pharmacokinetics via dosing interval. The results show that the system is responsive to these small changes, but does not fall apart due to them.

2) *On page 5, while beta is indeed the formal statistical mechanics way of naming this parameter, in practice describing its reciprocal as either kT (if using per particle energy units) or RT (if using molar units) would make the paper more accessible.*

We have added an additional definition of beta in the main text outside of the caption of Table 2 on page 5 to reiterate its definition and we also mention the alternative form 1/RT. We hope this finds a good balance between improving the accessibility and maintaining the statistical mechanics standard notation.

3) *On page 6, is the "total weight" the same thing as the statistical mechanical partition function? If so please state this, if not then a clear explanation of the difference would be valuable.*

We have added the following sentences to clarify this in an accessible way: "The probability of finding an enzyme in a particular state is the weight of that state divided by the sum of all the weights present in the system. In this way, the total weight of this system is the partition function of a single enzyme."

4) *On page 9, the general reader might infer that "dodecahedron" means the perfect Platonic solid of that name. Since this does not tessellate to fill three-dimensional Euclidean space, the authors probably have in mind some less regular dodecahedron such as the "rhombic dodecahedron" sometimes used as a simulation box where periodic boundary conditions are applied.*

This was a rhombic dodecahedron, and the change has been made.

5) *Again on page 9, a brief explanation or justification should be provided for the assumption about the sign of the I->A free energy difference.*

There are several statements in the text that provide an explanation: "It is assumed that the WT enzyme would prefer active state over inactive state, i.e. $\Delta G(I \rightarrow A)_{WT} < 0$." in combination with our definition of "The free-energy difference between the active and inactive enzymes states is denoted as $\Delta G(I \rightarrow A)$, which is defined as $\epsilon A - \epsilon I$ where $\epsilon A,I$ are the free energies of the active and inactive state of the enzyme, respectively.". However, we see that this is not very clear. To tie these ideas together more clearly across the text we have reiterated the previous statements when the next step is introduced.

6) *At various points in the manuscript, in-text citations of references or equations are out of numerical order, for example "[60, 23, 32]" is seen on page 3 and "Equations 11 and 10" appears on page 8.*

We have reviewed our references and citations and corrected those out of order.

Reviewer 3

1) *The motivation for developing the model felt unclear to me after reading the introduction and section 2. Although there are other examples, I highlight three.*

- *Example 1: "Many factors have been examined in the pursuit of finding the best choice of Abl1 inhibitor to switch to once resistant mutations arise." - This statement should have a list of citations for different methods – they only mention safety and tolerance.*

  Citations that support this statement have been included.

- *Example 2: "which mutations a drug is most effective against, rather than which drug is best suited to treating each mutation." - I don't understand the distinction. Can one not be informed by the other?*

  This statement has been changed to "which mutations each drug is most effective against, rather than which drug is best suited to treating each mutation." to better highlight the difference.

- *Example 3: "mutations that give rise to the mutant Abl1 enzymes affect other processes in the cell." – such as what? The authors allude to kcat not being a critical factor but it is not clear what else*

they are implying matters or how their model captures these other factors affected by a mutation. Are they just referring to the fact that Km might also be affected? I am not sure this qualifies as "other processes in the cell"

We have revised this statement to better emphasise that this is the keystone mechanism and that the catalytic values may indicate increased product formation rate, but could be ultimately useless information if, for example, the mutation provides an inactive enzyme state that is much more stable and meaning the likelihood of the substrate binding is very small.

We have also included more context for this work with a few sentences regarding current aims in CML treatment and research - specifically the aim of treatment free remission - and made other additional citations in the introduction and section 2.

2) *It would seem to make sense to me to put the final mass balance equations for their model (e.g. the one simulated to generated Figure 4) in a supplementary table as a compact representation of the model.*

We have provided this in the supplementary materials.

3) *Introduction: It seems like there should be more discussion of prior art on alternative metrics to IC50 or drawbacks of IC50 e.g. https://pubmed.ncbi.nlm.nih.gov/30562549/*

We have added more details and citations in this area including the suggested paper among others.

4) *Discussion: IC50s are used in many different fields – it would be nice if the authors could discuss further applications of these types of expanded dynamic models as alternatives to IC50s outside of this specific case.*

We have emphasised the scope of IC50 values in the discussion.

5) *Parameter sensitivity: Is the superiority of IRP over IC50 at all dependent on the model parameters?*

No, the IRP superiority over IC50 rises from the mimicry of patient conditions, not the model's parameters. We have now emphasised this point in our discussion.

6) *At the end of the day, is this model not more explanatory than predictive, since the IRP metric was presumably developed to match the measured resistance on the left side of Figure 6? That would not be a problem in itself, but I am curious what the practical utility of the model is in terms of informing treatment decisions. For example, in the Discussion the authors suggest: "This indicates that values of the average plasma concentrations of Abl1 inhibitors in patients combined with IC50 information could inform oncologists when selecting which second or third generation treatment to switch to once resistance arises and the mutation has been identified." Could the authors be even more specific and discuss hypothetical ranges where certain drugs would be selected under the status quo vs other drugs using the IRP-based criteria? i.e. can this model be converted into specific recommendations and potential impact assessed?*

IRP measurements from experiments or models that reflect patient concentrations could be used to predict resistance; however we realised these may not be attainable. We have emphasised this point in the final part of the discussion. We offer as an alternative, the other part of our work - effective IC50 ratio. To give an example of the usefulness of this ratio, in the results, we have described a potential example of misleading relative IC50 values for the mutation E255K, this should emphasise how the relative IC50 and effective IC50 ratio differ in a way that may not be obvious to all by examining what is now figure 7. We revisit this same example in the discussion with specific values.

7) *The title does not seem to reflect the focus of the Abstract – the title seems more general and the abstract more applied to a specific case. I would suggest better aligning them, maybe by instead presenting in the abstract the critical issues motivating the methodological developments in the work*

We have re-worked the abstract to better reflect the title and the content of the manuscript.

8) *There are a lot of figures that are individually not terribly informative or are repetitive – it seems like many of them could be combined (e.g. figures 2,3,4 and 9,10).*

We have combined the figures previously numbered 2-4 to give a sample of results for imatinib in the main text and included similar result samples for ponatinib and dasatinib in the supplementary materials. We have combined those labelled 9 and 10 into two sub figures together to emphasise their comparison.

9) *From Figure 1 it appears that the inhibitor binds equally well to the active and inactive form of the enzyme (uses the same parameters for each binding step). However, the authors state that "Imatinib and ponatinib both bind only to the inactive enzyme state while dasatinib binds more strongly to the active enzyme state." – thus it appears that these parameters are actually different based on the drug and potentially mutation? It would be nice if these details were more clear from the figure, and the quoted statement should have a citation as well.*

We have split figure one into two to better emphasise the difference between the system with imatinib or ponatinib and dasatinib. The phrase "dasatinib binds more strongly to the active enzyme state" has been revised after reviewing more information and we include further citations that evidence this statement (also see the response to Reviewer 1's first comment). We have also emphasised in the caption of this figure that it is a cartoon of the system and that the size of the rates between states differ depending on which mutation and inhibitor are present in the system.

Outside of the reviewers comments, we have also corrected typos, spelling and grammar errors, and replaced data that was mis-copied into the original (specifically Table 6).

We thank you again for your consideration.

Sincerely,

The authors
