# Supplementary material for: Beyond IC50—A computational dynamic model of drug resistance in enzyme inhibition treatment
Source: PLoS Comput Biol. 2024 Nov 7;20(11):e1012570. doi: 10.1371/journal.pcbi.1012570 (PMC11575782; doi:10.1371/journal.pcbi.1012570)
Supplement: Attachment — Submitted filename: resubmissionletter3.pdf [file pcbi.1012570.s005.pdf]

Dear editor and reviewers,

Thank you for your consideration and feedback. Below we reply to the last two points raised by Reviewer 1. In addition, we have also added an acknowledgement for computational resources.

Sincerely,

The authors.

Response to reviewer comments

Reviewer comment: *In my earlier comments I stated in error that G250E, E255K and E255V may favor (or bias towards) the inactive conformation where I meant to say the active state may be favored. I hope this did not result in any changes.*

We appreciate the reviwer realising this error. We only added a clarification in our previous revision, but did not make any other significant changes, so this has not resulted in any errors.

Revier comment: *On another front in looking at the protocol of the MD simulations to calculate free energies I am wondering if it would be more appropriate to have run these simulations in the presence of ATP. The data in table suggests equal or more favorable kcat with most mutations which could be due to better ATP binding. The authors should at least address this question or state why this was not done (and perhaps could be looked at in the future)*

The enzymes were modelled without bound ATP in the simulations since we assume that only the active site binds ATP. We have added a sentence to the manuscript explaining this.
